# Immunogenicity evaluation of recombinant rabies virus-vectored multivalent vaccines expressing feline calicivirus VP1 and feline herpesvirus 1 gB/gD genes

**DOI:** 10.1080/21505594.2026.2728426

**Published:** 2026-09-07

**Authors:** Yang Wang, Yueze Wang, Songze Zhao, Hongling He, Xiaolong Li, Yinyi Liang, Jiahao Wang, Lerong Xin, Nuan Chen, Wanxing Xie, Shile Huang, Jun Luo, Xiaofeng Guo

**Affiliations:** a College of Veterinary Medicine, South China Agricultural University, Guangzhou, China; b Department of Biochemistry and Molecular Biology, Louisiana State University Health Sciences Center, Shreveport, LA, USA; c Department of Hematology and Oncology, Louisiana State University Health Sciences Center, Shreveport, LA, USA; d Feist-Weiller Cancer Center, Louisiana State University Health Sciences Center, Shreveport, LA, USA

**Keywords:** Rabies virus, feline calicivirus, feline herpesvirus 1, vaccine, neutralizing antibody

## Abstract

Feline calicivirus (FCV) and feline herpesvirus 1 (FHV-1) are major pathogens threatening feline health, and prophylactic immunity remains the primary preventive approach. To develop a safe and effective multivalent vaccine against the two viruses, two recombinant rabies virus (RABV) vaccines expressing the FCV VP1 and FHV-1 gB/gD genes were constructed. The exogenous genes showed stable inheritance following serial viral passages, and the recombinant RABVs exhibited growth kinetics comparable to the parental strain, with a delayed peak titer. After verifying the viral pathogenicity, the recombinant RABV vaccines elicited specific IgG antibodies against VP1, gB, and gD, as well as neutralizing antibodies against FCV, FHV-1, and RABV in mice. Immunized mice were also protected against lethal RABV challenge. In cats, the vaccines induced neutralizing antibodies against the three pathogens, and immunized cats showed milder clinical signs and less severe histopathological lesions following FHV-1 and FCV challenge. Collectively, these findings indicate that the two recombinant vaccines induce robust humoral immune responses against FCV, FHV-1, and RABV in both mice and cats and confer protection against viral challenges.

## Introduction

Feline calicivirus (FCV) belongs to the genus *Vesivirus* within the family Caliciviridae. FCV primarily infects felids, including domestic cats, lions, tigers, and leopards [1]. Infection typically presents with clinical signs such as oral ulcerations, gingivostomatitis, hypersalivation, coughing, and conjunctivitis. Kittens are particularly susceptible, as infections often progress to fatal pneumonia [2]. The FCV capsid is composed of structural proteins VP1 and VP2. VP1 is critical for viral invasion and elicits neutralizing antibodies against FCV [3,4]. FCV is transmitted horizontally through saliva, ocular and nasal discharge from infected animals [5]. Notably, infected cats may shed the virus persistently even after clinical recovery, enabling ongoing transmission within populations [6]. Currently, clinically available FCV vaccines are predominantly attenuated or inactivated. However, preventing FCV infection remains challenging due to extensive strain diversity and limited heterologous cross-protection [7]. Epidemiological surveillance has revealed substantial genetic variability in *VP1* between currently circulating strains and vaccine strains [8], highlighting the need for vaccines targeting emerging variants.

Feline viral rhinotracheitis (FVR) is an acute and highly contagious upper respiratory tract disease caused by feline herpesvirus 1 (FHV-1). This virus primarily infects felids, including domestic cats, tigers, lions, and cheetahs [9]. The most common clinical signs of FVR include sneezing, keratoconjunctivitis, and serous ocular and nasal discharge [10,11]. FHV-1 establishes latency in the host’s trigeminal ganglia, leading to lifelong infection, and the latent virus can reactivate upon immune suppression or external stimuli [12,13]. Vaccination is the primary means of preventing FVR. Both inactivated and modified live virus vaccines currently available provide effective protection [14]. Additionally, subunit vaccines based on viral glycoproteins and delivered using bacterial-like particles as vectors are under investigation [15]. Several studies have developed bivalent vaccines by substituting virulence-associated genes of FHV-1 with genes such as FCV *VP1*, feline parvovirus (FPV) *VP2*, or RABV *G* [16–18], reflecting recent advances in the development of novel FHV-1 vaccines.

Rabies is a zoonotic infectious disease caused by rabies virus (RABV), with a mortality rate approaching 100% after the onset of clinical symptoms in infected humans and other susceptible mammals [19]. The majority of rabies cases in China result from dog bites or scratches, but some cases are attributed to cats [20]. Therefore, rabies prevention for cats is also of critical importance for overall disease control. As this primarily relies on vaccination, the development of safe and effective feline rabies vaccines is indispensable. Notably, RABV has emerged as a promising recombinant vaccine vector owing to its capacity for large scale *in vitro* culture and efficient exogenous protein expression. Recent studies have extensively explored RABV as a vector for vaccines against various pathogens, including severe acute respiratory syndrome coronavirus 2 [21,22], Marburg virus [23], Rift Valley fever virus [24], and human immunodeficiency virus [25]. Importantly, recombinant RABV vaccines elicit a robust immune response, supporting RABV’s significant potential as a viral vector for multivalent vaccine development.

In this study, we constructed trivalent inactivated vaccines by inserting FCV *VP1*, FHV-1 *gD*, and FHV-1 *gB* genes individually into the RABV genome and evaluated their immunogenicity. The results demonstrated that these recombinant RABV vaccines elicited specific IgG antibodies against VP1, gD, and gB, as well as neutralizing antibodies against FCV, FHV-1, and RABV in both mice and cats, while exhibiting protective efficacy against these viruses’ challenge. By reducing the number of required vaccinations while conferring simultaneous protection against FCV, FHV-1, and RABV, the novel recombinant trivalent vaccines represent a significant advancement in the prevention and control of common feline infectious diseases.

## Materials and methods

### Cells, plasmids, viruses, reagents, and animals

Baby hamster kidney (BHK-21) cells, feline kidney (F81) cells and mouse neuroblastoma (NA) cells were purchased from Wuhan Institute of Biological Products (Wuhan, Hubei, China). BHK-21 and F81 cells were maintained in Dulbecco’s Modified Eagle’s Medium (DMEM) (Gibco, Grand Island, NY, USA), while NA cells were cultured in Roswell Park Memorial Institute (RPMI) 1640 medium (Gibco). Both media were supplemented with 10% fetal bovine serum (Gibco). The RABV challenge virus standard strain CVS-11 was kindly provided by Dr. Xianzhu Xia (Academy of Military Medical Sciences, Beijing, China) and propagated in NA cells. The recombinant RABV strain rHEP-Flury, derived from the vaccine strain HEP-Flury, was rescued using the following plasmids: pHEP-3.0, pCDNA3.1-N, pCDNA3.1-P, pCDNA3.1-G, and pCDNA3.1-L, which were kindly provided by Dr. Kinjiro Morimoto (Yasuda Women’s University, Hiroshima, Japan). The pHEP-NP-GFP infectious clone, constructed in our previous study [26], was used as a positive control. Mouse anti-FCV VP1 polyclonal sera, mouse anti-FHV-1 gB polyclonal sera, mouse anti-FHV-1 gD polyclonal sera, and mouse anti-RABV-M monoclonal antibody were prepared in the laboratory (unpublished data). High-fidelity DNA polymerase (P521), a seamless cloning kit (C112), and Ni-NTA agarose resin (HP201) were obtained from Vazyme Biotech Co., Ltd. (Nanjing, Jiangsu, China). Horseradish peroxidase (HRP)-conjugated goat anti-mouse IgG antibody (SA00001-1) and fluorescein isothiocyanate (FITC)-conjugated goat anti-mouse IgG antibody (SA00003-1) were purchased from Proteintech (Wuhan, Hubei, China). Trypsin-EDTA (0.25%) was purchased from Gibco. Penicillin-streptomycin mixture was obtained from Biosharp Life Sciences (Hefei, Anhui, China). Transfection reagent Lipofectamine^TM^ 3000 (L3000015) and restriction endonucleases *BsiW*I (ER0851) and *Nhe*I (ER0972) were provided by Thermo Fisher Scientific Inc. (Waltham, MA, USA). FITC-conjugated anti-RABV N monoclonal antibody (800–092) was purchased from Fujirebio Inc. (Malvern, PA, USA). A total of 81 specific-pathogen-free (SPF) female Kunming (KM) and 9 SPF female BALB/c mice, aged 6–8 weeks, were obtained from Zhuhai Bestest Biotech Co., Ltd. (Zhuhai, Guangdong, China). A total of 24 Chinese domestic cats (8–10 weeks old) were purchased from Guangzhou Juchongmengmao Pet Co., Ltd. (Guangzhou, Guangdong, China). All animals were housed in the Laboratory Animal Center of South China Agricultural University (Guangzhou, Guangdong, China) under controlled environmental conditions, including a 12 h light-dark cycle and *ad libitum* access to food and water. All animal experiments were conducted in a biosafety level 2 laboratory and strictly complied with the ARRIVE Guidelines 2.0. The experimental protocol was reviewed and approved by the Animal Ethics and Use Committee of South China Agricultural University (Approval number: 2024c054, 2025c145, and 2025f399).

## Plasmid construction and virus rescue

PCR amplification of the *VP1*, *gB*, and *gD* genes was performed on the FCV strain GDJM-202201 (GenBank: OQ472996.1) and the FHV-1 strain SH-01c, both isolated by our laboratory. Primers are listed in Table S1. First, the amplified *VP1* gene product was cloned using infusion cloning between the *N* and *P* genes of pHEP3.0 to generate the plasmid pHEP3.0-VP1. During PCR amplification of pHEP3.0 (Primers are listed in Table S2), RABV-derived transcription initiation signal (TIS) and transcription termination signal (TTS) sequences were introduced downstream of the *N* gene and upstream of the *P* gene, respectively, to enable transcription of the inserted *VP1* gene. Next, the amplified FHV-1 *gD* and *gB* gene products were individually inserted between the *G* and *L* genes at the *BsiW*I and *Nhe*I restriction sites to obtain the plasmids pHEP3.0-VP1-gD and pHEP3.0-VP1-gB, respectively. The regions flanking the *BsiW*I and *Nhe*I restriction sites in pHEP3.0 already contained TIS and TTS sequences. The full-length antigenomic cDNA constructs were generated under the control of the cytomegalovirus (CMV) promoter. Virus rescue was performed as previously described [26]. Briefly, the plasmids pHEP3.0-VP1-gD and pHEP3.0-VP1-gB were co-transfected into BHK-21 cells along with helper plasmids. Cell supernatants were collected for direct immunofluorescence assay (DFA) on day 7 post-transfection, and DFA-positive supernatants were further passaged in BHK-21 cells.

## Immunofluorescence assay

Supernatants from the rescued virus were inoculated into NA cells seeded in tissue culture-treated 96-well plates at a volume of 35 μL per well, with 3 replicate wells per sample. The plates were stored in an incubator with 5% CO_2_ at 37°C. In 48 h, the used medium was discarded, and the cells were fixed for 30 min in 80% acetone at −20°C. Following fixation, the cells were washed 3 times with phosphate buffered saline (PBS), and 50 μL of FITC-conjugated anti-RABV N monoclonal antibody (diluted 1:500) was then added to each well. The plates were incubated overnight at 4°C and subsequently observed under a fluorescence microscope (AMG, Mill Creek, WA, USA).

The indirect immunofluorescence assay (IFA) procedure followed a protocol similar to that of the DFA. After fixation, the cells were incubated overnight at 4°C with polyclonal sera against FCV VP1 (diluted 1:500). The antibody solution was then discarded, and the cells were washed 3 times with PBS. Next, FITC-conjugated goat anti-mouse IgG antibody (diluted 1:500) was added to each well, and the plates were incubated for 30 min at 37°C in dark before observation under a fluorescence microscope. The IFA procedures for gB and gD were identical to that for VP1.

## Western blot analysis

The cell culture medium was discarded, and the cells were washed 3 times with PBS. Cells were lysed using cell lysis solution. An equal amount of protein was loaded into a sodium dodecyl sulfate-polyacrylamide gel for electrophoresis and subsequently transferred onto a polyvinylidene fluoride membrane. The membranes were blocked in 5% skimmed milk solution at room temperature for 1 h, and then incubated overnight at 4°C with the primary antibodies (anti-FCV VP1, anti-FHV-1 gB, or anti-FHV-1 gD) (all 1:1000 dilution). This was followed by incubation with HRP-conjugated goat anti-mouse IgG antibody (1:50,000 dilution) at room temperature for 1 h. The blots were visualized using the Fine-do X6 system exposure apparatus (Tanon, Shanghai, China).

## Preparation and characterization of polyclonal antibodies against FHV-1 gD/gB and FCV VP1

Partial fragments of the FHV-1 *gD* and *gB* genes, as well as the FCV *VP1* gene, were amplified by PCR using the primers listed in Table S3. The *gD* and *gB* fragments were cloned into the prokaryotic expression vector pET-32a, whereas the FCV *VP1* fragment was inserted into pET-28a. The recombinant plasmids were transformed into *Escherichia coli* BL21 competent cells for protein expression. Following induction with isopropyl β-D-1-thiogalactopyranoside, recombinant proteins were purified using Ni-NTA affinity chromatography. Purified proteins were emulsified with Freund’s complete adjuvant (Sigma-Aldrich, St. Louis, MO, USA, F5881) and used to immunize three BALB/c mice, followed by two booster immunizations at 2-week intervals with Freund’s incomplete adjuvant (Sigma-Aldrich, F5506). Retro-orbital blood samples were collected 14 days after the second booster immunization, and sera were isolated for use as polyclonal antibodies. Antibody specificity was validated by Western blot analysis using lysates from virus-infected cells (Figure S1).

## Enzyme-linked immunosorbent assay (ELISA) for IgG detection

The purified VP1, gD, or gB proteins expressed in a prokaryotic system were individually coated onto flat-bottom 96-well ELISA plates (BIOFIL, Guangzhou, Guangdong, China; FEP101896) at a concentration of 10 μg/mL (100 μL/well) and incubated overnight at 4°C. After removal of the coating solution, 100 μL of 5% bovine serum albumin in 1 × PBS was added to each well for blocking at 37°C for 1 h. The plates were then washed 3 times with PBS containing 0.1% Tween 20 (PBST), with each wash performed by shaking the plates in PBST for 5–10 min. Serum samples were serially diluted 2-fold, and 100 μL of each diluted sample was added to the wells. The plates were incubated at 37°C for 1 h, followed by 3 washes with PBST as described above. Next, 100 μL of HRP-conjugated goat anti-mouse IgG antibody (diluted 1:10,000) was added to each well, and the plates were incubated at 37°C for 40 min. After washing the wells 3 times with PBST as described above, 100 μL of tetramethylbenzidine (TMB) solution (Beyotime, P0209) was added to each well. After incubation at 37°C for 10 min, 50 μL of 2 M H_2_SO_4_ solution was added to each well. Finally, the absorbance at 450 nm was measured using a microplate reader (Bio-Rad, Hercules, CA, USA). Serum samples from 3 biologically independent animals were analyzed for each experimental group.

## Growth curve analysis of recombinant RABVs

BHK-21 cells were seeded into 100-mm dishes at a density of 1 × 10^6^ cells/dish. Once the cells reached 70–80% confluence, the used medium was aspirated. After washing 3 times with PBS, the cells were inoculated with rHEP-VP1-gB, rHEP-VP1-gD, or rHEP-Flury at a multiplicity of infection (MOI) of 1 or 0.1. Following incubation for 1 h at 37°C, the culture supernatants were removed, and the cells were washed 3 times with PBS. Next, 10 mL of fresh medium was added to each dish. Subsequently, 200 μL of culture supernatants was collected from each dish every 24 h, and 200 μL of fresh medium was added back to each dish. All collected supernatants were stored at −80°C until viral titer detection.

One day before viral titer detection, NA cells were seeded into 96-well plates at a density of 1 × 10^4^ cells/well. When the cells reached 70–80% confluence, the culture supernatants were serially diluted 10-fold with RPMI 1640 medium. Each dilution was inoculated into 4 replicate wells (100 μL/well). The plates were incubated at 37°C with 5% CO_2_ for 2 days before the cells underwent DFA. Viral titers were calculated as focus-forming units per milliliter (FFU/mL) using the Karber method.

## Pathogenicity assessment of recombinant RABVs

6-week-old KM mice were randomly divided into two independent cohorts. In the first cohort (*n* = 5 per group, except for the rHEP-VP1-gD group, *n* = 6), mice were intracranially inoculated with 30 μL of rHEP-VP1-gB, rHEP-VP1-gD, rHEP-Flury, or CVS-11 (10^6.5^ FFU/mL), while control mice received 30 μL of DMEM via the same route. Body weight was recorded daily for 17 days post-inoculation, and survival was monitored for up to 25 days. In the second cohort (*n* = 3 per group), mice received the same inoculation procedures and doses and were used for histopathological analysis. At 15 days post-inoculation, mice were perfused and brain tissues were collected for histopathological examination.

## Recombinant RABV vaccine preparation and animal inoculation

The rHEP-VP1-gB, rHEP-VP1-gD, and rHEP-Flury were inactivated with betapropiolactone at a ratio of 2000:1 and incubated for 24 h at 4°C, followed by incubation at 37°C for 2 h to facilitate hydrolysis of residual betapropiolactone. Next, the inactivated viruses were mixed with MONTANIDE GEL adjuvant (SEPPIC, France) at a ratio of 9:1, and the mixtures were stirred at 4°C using a magnetic stirrer until stable vaccine formulations without phase separation were obtained.

Forty 7-week-old female KM mice were randomly assigned to 4 groups (10 mice/group), including the rHEP-VP1-gB vaccine group, rHEP-VP1-gD vaccine group, rHEP-Flury vaccine group, and DMEM control group. Each mouse was injected intramuscularly with 100 μL of the corresponding vaccine formulation (10^6.5^ FFU/mL). The control group received 100 μL of DMEM mixed with the adjuvant. Body weight was measured daily for 14 days following the initial immunization. Booster doses were administered on days 14 and 28 post-initial immunization, and blood samples were collected on days 7, 21, 35, 42, 49, and 56 post-initial immunization. For all mouse experiments, euthanasia was performed either at the conclusion of each experiment or when mice manifested signs of coma, paralysis, or immobility despite provocation. The procedure was carried out via intraperitoneal injection of 5% sodium pentobarbital solution (150–200 mg/kg) in accordance with the Chinese Guidelines for the Euthanasia of Laboratory Animals. Death was confirmed by concurrent cessation of respiration and cardiac activity, loss of the corneal reflex, and limb muscle flaccidity.

Cats negative for FCV and FHV-1 by PCR (Primers are listed in Table S4) and with FCV and FHV-1 neutralizing antibody titers below 1:2 were selected for the study. Twenty-four Chinese domestic cats (8–10 weeks old) were randomly divided into 4 groups (6 cats/group), including the rHEP-VP1-gB vaccine group, rHEP-VP1-gD vaccine group, rHEP-Flury vaccine group, and DMEM control group. Each cat was injected subcutaneously with 1 mL of the corresponding vaccine formulation (10^8^ FFU/mL). The control group received 1 mL of DMEM mixed with the adjuvant. Booster immunizations were administered on days 14 and 28 post-initial immunization. Blood samples were collected from the hindlimb veins on days 7, 21, and 35 post-initial immunization.

## Neutralizing antibody assay

All serum samples were separated by centrifugation at 2000 rpm for 10 min and then heated at 56°C for 30 min. Subsequently, the serum samples were serially diluted 2-fold in DMEM in 96-well plates, with 3 replicate wells per sample. The samples were incubated with 50 μL of 100 TCID_50_ FCV-GDJM-202201 or FHV-1-SH-01c for 1 h at 37°C. Afterward, 2 × 10^4^ F81 cells were added to each well, and the plates were incubated at 37°C with 5% CO_2_ for 48 h. Next, the plates were analyzed by IFA. Neutralization titers were determined by calculating the average of the highest dilution of the wells that showed no specific fluorescence after staining. The procedure for detecting neutralizing antibodies against RABV was similar, with specific steps detailed in a previous study [27].

## Challenge assay

On day 7 post the third immunization, 5 mice were randomly selected from each group. Each mouse was inoculated intramuscularly with 100 μL of CVS-11 at a titer of 1 × 10^6.5^ FFU/mL. After the challenge, the mice were monitored daily for mortality. Additionally, daily weight measurements and clinical manifestation assessments were conducted. The scoring criteria for clinical manifestations were described previously [28].

On day 37 post the primary vaccination, 3 cats were randomly selected from each group and separately challenged with FCV-GDJM-202201 or FHV-1-SH-01c. For FCV challenge, each cat was intranasally administered 500 μL of FCV (10^8^ FFU/mL), with 250 μL delivered into each nostril. For FHV-1 challenge, each cat received both intranasal and ocular inoculations totaling 1 mL (10^6.5^ FFU/mL), with 200 μL administered into each nostril and 300 μL instilled dropwise into each eye. Monitoring of body weight/temperature, clinical signs, and mortality was conducted for 14 days post-challenge. The clinical scoring criteria for FHV-1 are presented in Table 1, and those for FCV are detailed in Table 2. Additionally, swabs were collected from each cat at each monitoring time point. RNA extracted from swabs collected from cats in the FCV challenge group was reverse-transcribed into cDNA, while DNA was extracted from swabs collected from cats in the FHV-1 challenge group. Viral loads were quantified by quantitative real-time PCR (qPCR) using primers listed in Table S5. When cats exhibited coma, anorexia, or immobility despite provocation, they were humanely euthanized by intravenous injection of an overdose of 5% sodium pentobarbital solution (100–150 mg/kg) in accordance with the Chinese Guidelines for the Euthanasia of Laboratory Animals. Death was confirmed by the absence of vital signs and the observation of fixed, dilated pupils.

**Table 1.** Clinical scoring criteria for FHV-1.

Category	Signs	Score
Mental status	Normal	0
	Reduced activity	1
	Lethargic	2
Nasal discharge	Normal	0
	Minor, serous	1
	mucopurulent	2
Ocular discharge	Normal	0
	Minor, serous	1
	mucopurulent	2
Sneezing	Normal	0
	Observed	2
Coughing	Normal	0
	Observed	2
Conjunctivitis	Normal	0
	Edema, hyperemia	2
weight	Weight change >-5%	0
	Weight change ≤ −5%	5

If an animal died during the observation period, it would be assigned a clinical score of 10 points.

**Table 2.** Clinical scoring criteria for FCV.

Category	Signs	Score
Mental status	Normal	0
	Reduced activity	1
	Lethargic	2
Dehydration	Normal	0
	slight skin tenting (≤3 s)	1
	persistent skin tenting (>3 s)	2
Ocular discharge	Normal	0
	Minor, serous	1
	mucopurulent	2
Nasal discharge	Normal	0
	Minor, serous	1
	mucopurulent	2
Ulceration	Normal	0
	Observed	2
weight	Weight change >-5%	0
	Weight change ≤ −5%	5

If an animal died during the observation period, it would be assigned a clinical score of 10 points.

## Histopathological analysis

Lung and trachea samples were obtained after the 14-day observation period following the challenge assays. Briefly, the tissues were fixed in 4% paraformaldehyde solution for 24 h. Next, the tissues underwent dehydration, clearing, and embedding in paraffin. Sections were cut to a thickness of 4–5 μm and stained using hematoxylin and eosin (H&E).

## Data analysis

Results were presented as mean ± standard deviation (SD). GraphPad Prism 8 was used for statistical analysis. Statistical significance was assessed using unpaired *t*-tests or two-way ANOVA followed by Tukey’s multiple comparison test. Differences with *p* values < 0.05 were considered statistically significant.

## Results

### Rescue of recombinant RABVs

**Figure 1.**
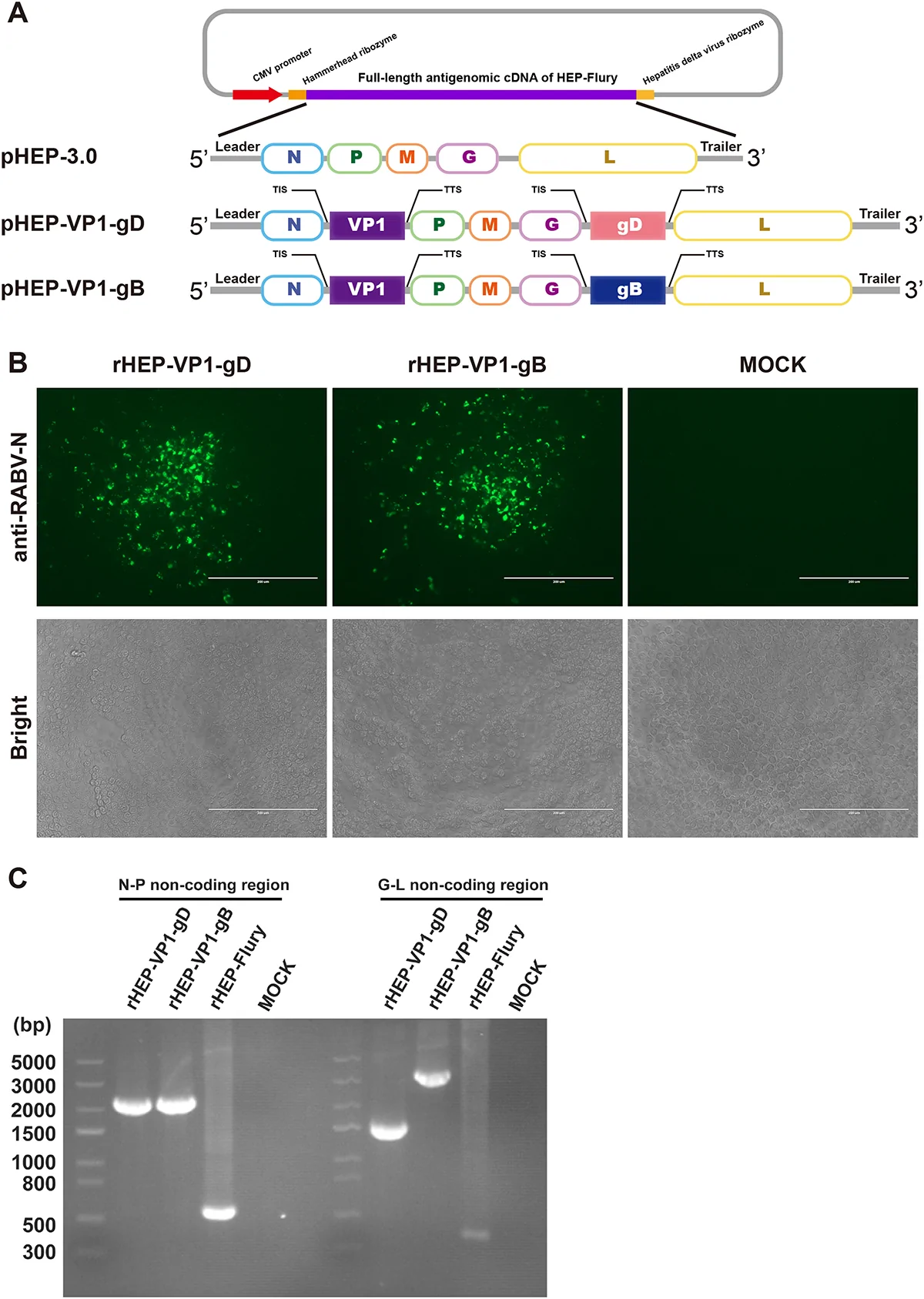
Rescue and identification of recombinant RABV. (A) schematic representation of the full-length infectious clone plasmids of rHEP-Flury and the recombinant RABVs. (B) supernatants from BHK-21 cells co-transfected with plasmids were collected and inoculated into NA cells. After incubation, the cells were fixed and incubated with the FITC-conjugated monoclonal antibody anti-RABV N overnight, followed by observation under a fluorescence microscope. Scale bar: 200 μm. (C) RNA was extracted from the rescued viruses and reverse-transcribed into cDNA. The non-coding regions between the *N-P* and *G-L* genes of viral genome were amplified by PCR.

The *VP1* gene was amplified from the FCV-GDJM-202201 isolate, while the *gB* and *gD* genes were amplified from the FHV-1-SH-01c isolate. The *VP1* gene was first cloned into the rHEP-Flury vector between the *N* and *P* genes. Subsequently, the *gB* or *gD* genes were individually cloned into the rHEP-VP1 vector between the *G* and *L* genes using restriction sites Figure 1(A). The full-length viral cDNA plasmids, along with helper plasmids, were co-transfected into BHK-21 cells to rescue two recombinant RABVs, designated as rHEP-VP1-gB and rHEP-VP1-gD. Infection of both recombinant RABVs was confirmed by DFA assay, which revealed specific green fluorescent foci in infected cells Figure 1(B). Additionally, the inserted exogenous genes were successfully amplified from both recombinant RABVs by reverse transcription PCR (RT-PCR) Figure 1(C).

## Characterization of exogenous protein expression

**Figure 2.**
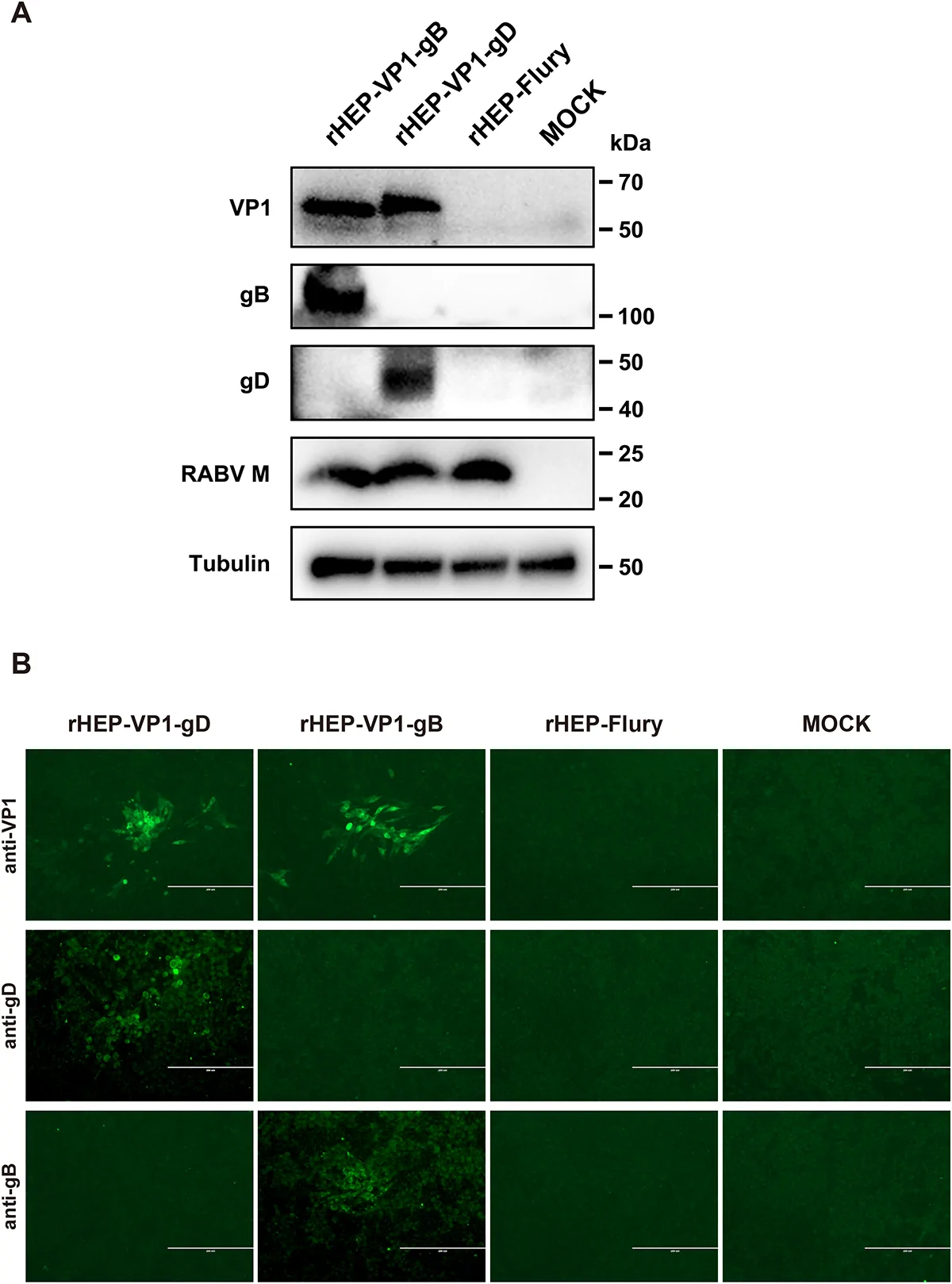
Identification of exogenous gene expression. (A) recombinant RABVs were inoculated into BHK-21 cells, and protein samples were collected at 5 days post-infection, followed by Western blot analysis using the indicated antibodies. (B) following a similar infection procedure, recombinant RABVs were inoculated into BHK-21 cells. IFA was performed with indicated antibodies. Scale bar: 200 μm.

To determine whether the exogenous genes were effectively expressed, BHK-21 cells infected with rHEP-VP1-gB or rHEP-VP1-gD were analyzed using Western blot and IFA. The expression of VP1 and gB proteins was confirmed in the cells infected with rHEP-VP1-gB, while the expression of VP1 and gD proteins was confirmed in the cells infected with rHEP-VP1-gD Figure 2(A). Subsequently, IFA was performed to further confirm the expression of the exogenous proteins. Distinct green fluorescent foci were observed in cells infected with rHEP-VP1-gB or rHEP-VP1-gD upon staining with antibodies against VP1, gD, or gB, respectively Figure 2(B).

## Assessment of the genetic stability and growth kinetics of recombinant RABVs

**Figure 3.**
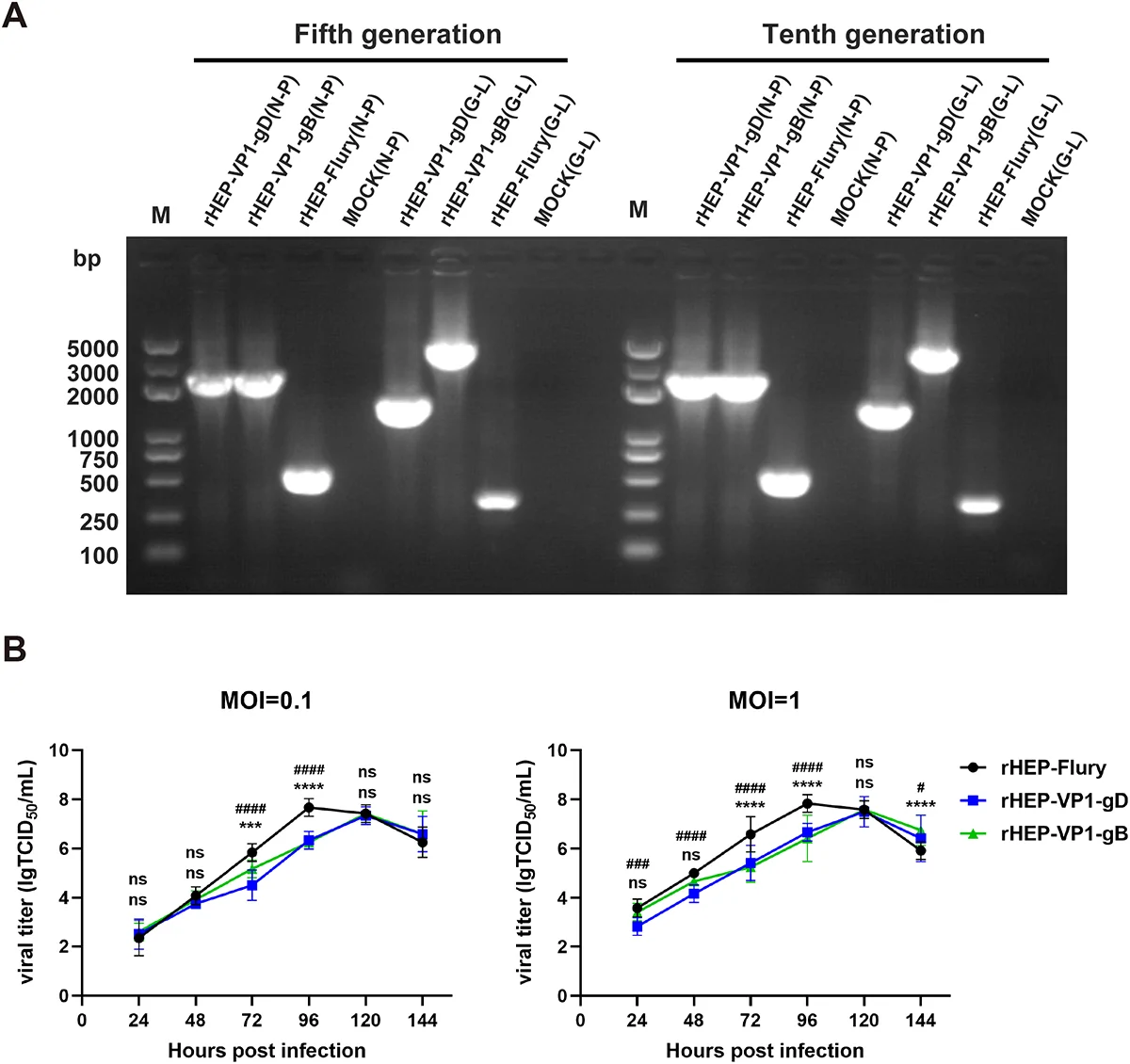
Assessment of genetic stability of exogenous genes and viral growth kinetics in BHK-21 cells. (A) RNA was extracted from the viral supernatants of the 5th and 10th passages and reverse-transcribed into cDNA. PCR amplification was performed using primer pairs located within the *N* and *P* genes, or within the *G* and *L* genes, to flank the exogenous gene insertion sites. (B) recombinant RABVs were inoculated into BHK-21 cells at an MOI of 1 or 0.1. Cell supernatants were collected every 24 h post-inoculation to determine viral titers. Corresponding viral growth curves were plotted using GraphPad Prism software. Data are presented as mean ± SD (*n* = 3). Statistical analyses were performed using two-way ANOVA. # statistical significance between the rHEP-VP1-gD and rHEP-Flury groups; * statistical significance between the rHEP-VP1-gB and rHEP-Flury groups (^#^or*p < 0.05; ^##^or**p < 0.01; ^###^or***p < 0.001; ^####^or****p < 0.0001; ns, not significant).

To evaluate the genetic stability of the inserted exogenous genes during serial *in vitro* passage, viral RNA was extracted from the 5th and 10th passages of rHEP-VP1-gB and rHEP-VP1-gD, as well as the parental strain rHEP-Flury. cDNA was synthesized, and PCR amplification was performed using primers flanking the insertion sites (Table S6). PCR products from rHEP-VP1-gB, rHEP-VP1-gD, and rHEP-Flury were obtained Figure 3(A). Both recombinant RABVs exhibited larger amplicon sizes compared with those of rHEP-Flury, consistent with successful insertion of the exogenous genes. The resulting PCR products were further subjected to Sanger sequencing analysis. The sequencing results confirmed that the inserted exogenous genes in both rHEP-VP1-gB and rHEP-VP1-gD remained intact and free of detectable mutations after 10 serial passages.

To investigate the growth kinetics of rHEP-VP1-gB and rHEP-VP1-gD, BHK-21 cells were infected with each virus at an MOI of 1 or 0.1, with rHEP-Flury used as the parental control. The results showed that the growth curves of rHEP-VP1-gB and rHEP-VP1-gD exhibited comparable overall profiles to those of rHEP-Flury. Under both synchronous and asynchronous infection conditions, rHEP-VP1-gB and rHEP-VP1-gD reached peak titers at 120 h post-infection, followed by a decline until 144 h. In contrast, rHEP-Flury reached its peak titer at 96 h post-infection, followed by a decline over time. Notably, the peak viral titers of rHEP-VP1-gB and rHEP-VP1-gD were comparable to those of rHEP-Flury Figure 3(B).

## Pathogenicity of recombinant RABVs

**Figure 4.**
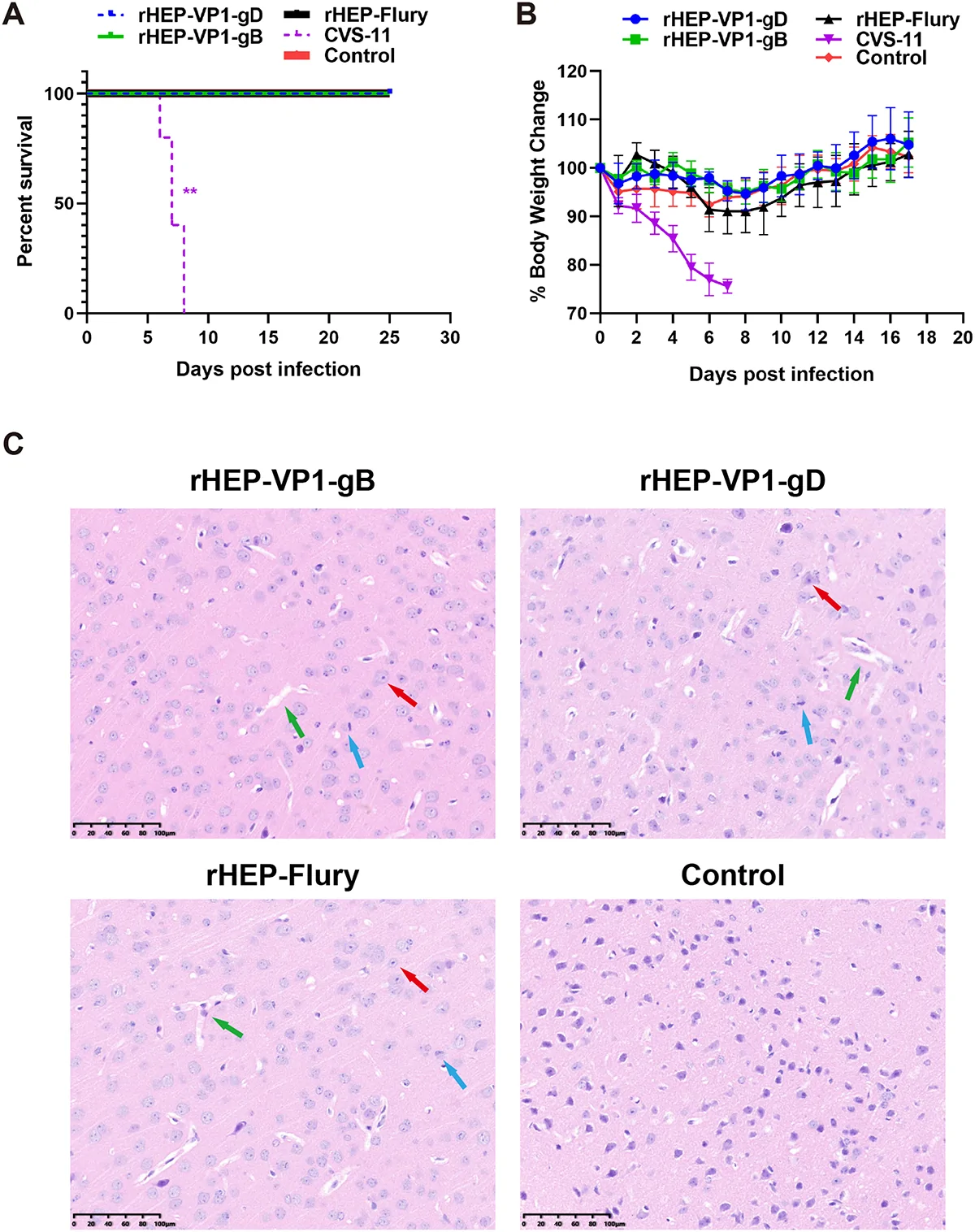
Pathogenicity of recombinant RABVs in mice. (A) 30 μL of recombinant RABV suspension (10^6.5^ FFU/mL) was intracranially inoculated into 6-week-old KM mice (*n* = 5 per group, except for the rHEP-VP1-gD group, *n* = 6). Mortality was monitored continuously for 25 days post-inoculation, and survival curves were statistically analyzed using the log-rank mantel-cox test (*p < 0.05; **p < 0.01). (B) body weight was recorded daily for 17 consecutive days post-infection. (C) brain tissue sections were prepared from mice infected with recombinant RABVs, and representative images are shown. Scale bar: 100 μm. Data are presented as mean ± SD.

To evaluate the pathogenicity of the recombinant RABVs, 6-week-old KM mice were intracranially inoculated with rHEP-VP1-gB, rHEP-VP1-gD, rHEP-Flury, or CVS-11. Mice infected with CVS-11 began to die 6 days post-infection, with all succumbing by day 8. In contrast, all mice infected with the recombinant RABVs or rHEP-Flury survived up to 25 days post-infection Figure 4(A). Analysis of body weight changes revealed that mice infected with CVS-11 experienced a continuous and significant weight loss from day 1 until death. Conversely, mice infected with the recombinant RABVs or rHEP-Flury exhibited transient body weight loss, after which their body weights gradually recovered and increased Figure 4(B). Histopathological analysis of the cerebral cortex in mice infected with the recombinant RABVs or rHEP-Flury revealed numerous loose interstitial spaces (indicated by green arrows). Additionally, nuclear pyknosis and displacement were observed in a subset of neurons (highlighted with red and blue arrows, respectively) Figure 4(C). These results indicate that both recombinant RABVs induce only mild pathological lesions in mouse brain tissue, primarily characterized by neuronal degeneration, with pathogenicity comparable to that of rHEP-Flury.

## Immunogenicity of recombinant RABV vaccines in mice

**Figure 5.**
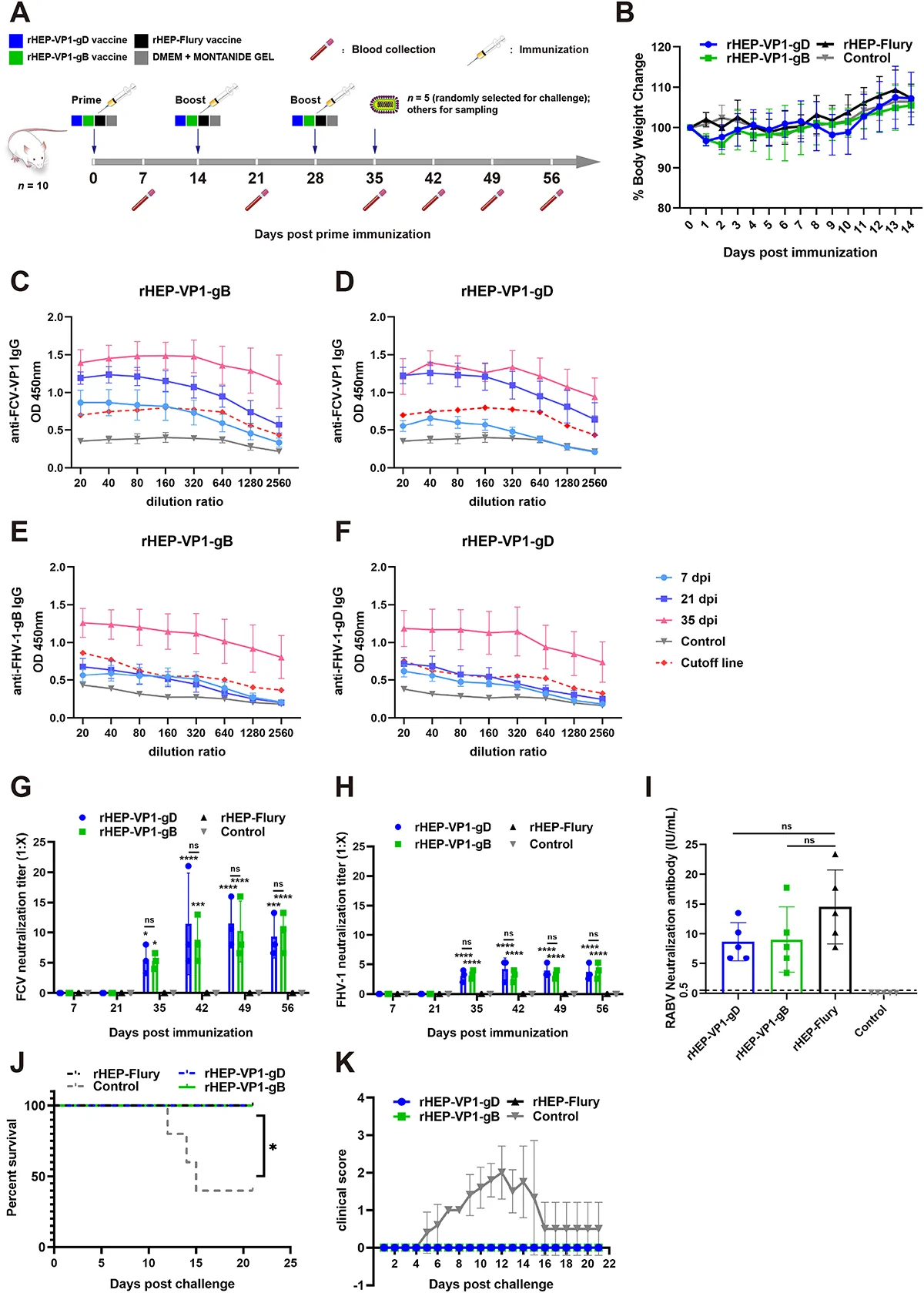
Evaluation of immunogenicity of the recombinant RABV vaccines in mice. (A) 7-week-old KM mice were intramuscularly inoculated with 100 μL of recombinant RABV vaccines (10^6.5^ FFU/mL), followed by booster immunizations at 14 and 28 days post-initial immunization. Blood samples were collected on days 7, 21, 35, 42, 49, and 56. (B) mice were weighed daily for 14 consecutive days post-initial immunization. (C-F) serum samples were randomly selected from each group at 7 days post each immunization (*n* = 3). ELISA was performed with 2-fold serially diluted serum samples to quantify the relative levels of anti-VP1, anti-gB, and anti-gD IgG antibodies. Sera from mice inoculated with DMEM and adjuvant served as the negative control. The cutoff value was defined as two times the mean OD_450_ value of the control sera. (G-I) serum neutralizing antibodies against FCV and FHV-1 were detected at 7, 21, 35, 42, 49, and 56 days post-immunization, while those against RABV were assayed specifically at 35 days post-immunization. (J) immunized mice were intramuscularly challenged with CVS-11 at a titer of 10^6.5^ FFU/mL, mortality was monitored for 21 consecutive days post-challenge. (K) following the challenge, clinical signs were assessed daily for 21 consecutive days. Data are presented as mean ± SD (*n* = 3 or 5). Significant differences between groups were assessed using *t*-test (*p < 0.05; **p < 0.01; ***p < 0.001; ****p < 0.0001; ns, not significant).

To evaluate the immunogenicity of the recombinant RABV vaccines in mice, 7-week-old KM mice were immunized with rHEP-VP1-gB, rHEP-VP1-gD, or rHEP-Flury vaccines 3 times at 2-week intervals. Blood samples were collected on days 7, 21, 35, 42, 49, and 56 post-primary immunization to measure antibody levels Figure 5(A). Additionally, the body weights of the mice were recorded following the initial vaccination. The immunized mice exhibited a decrease in body weight on the first day, followed by a gradual increase. This trend was not significantly different from that observed in the rHEP-Flury group or the control group Figure 5(B). ELISA analysis revealed a gradual increase in antigen-specific IgG responses following immunization. VP1-specific IgG antibodies became clearly detectable above the cutoff value by day 21 post-immunization, whereas gB- and gD-specific IgG antibodies became clearly detectable above the cutoff value by day 35 Figure 5(C,F). Neutralizing antibodies against FCV and FHV-1 were detectable in the sera of mice 35 days post primary immunization. While the rHEP-VP1-gB and rHEP-VP1-gD vaccines showed no significant difference in neutralizing antibody titers against FCV, most mouse sera exhibited titers greater than 1:8, with some reaching titers greater than 1:16. Regarding neutralizing antibodies against FHV-1, no significant difference was observed between the rHEP-VP1-gB and rHEP-VP1-gD vaccines, with neutralizing titers ranging from 1:2 to 1:4 Figure 5(G,H). Furthermore, detection of RABV neutralizing antibodies indicated that by 35 days post-immunization, both rHEP-VP1-gB and rHEP-VP1-gD effectively induced neutralizing antibodies against RABV, with all samples showing titers greater than 0.5 IU/mL (the World Health Organization-recommended protective neutralizing antibody titer). Importantly, the levels of RABV neutralizing antibodies induced by the rHEP-VP1-gB or rHEP-VP1-gD vaccines did not significantly differ from those induced by rHEP-Flury vaccine Figure 5(I).

To evaluate the protective efficacy of the recombinant RABV vaccines against RABV, immunized mice were intramuscularly challenged with the virulent RABV strain CVS-11. The results showed that mice in the control group succumbed to infection between days 12 and 15 post-challenge, with a mortality rate of 60%. In contrast, mice immunized with the rHEP-VP1-gB or rHEP-VP1-gD vaccines exhibited no fatalities during the observation period Figure 5(J). Additionally, clinical performance scores indicated that the immunized mice showed no significant clinical symptoms following the lethal challenge, whereas control group mice began to display notable clinical signs on day 5 post-challenge, and some did not fully recover until the end of the observation period Figure 5(K).

## Immunogenicity of recombinant RABV vaccine in cats

**Figure 6.**
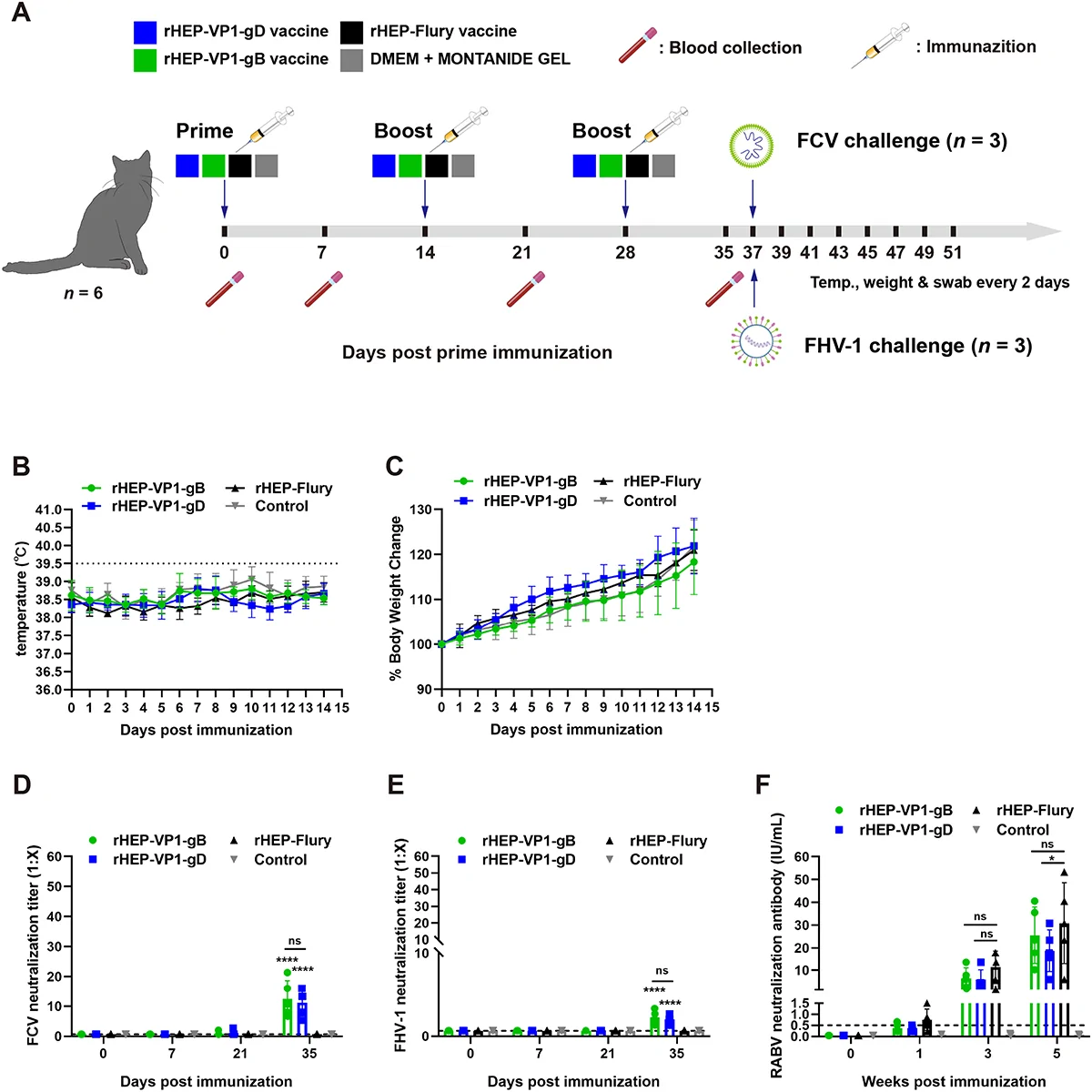
Evaluation of immunogenicity of recombinant RABV vaccines in cats. (A) recombinant RABV vaccines (1 mL, 10^8^ FFU/mL) were subcutaneously administered to cats on days 0, 14, and 28. Blood samples were collected on days 0, 7, 21, and 35 post-initial immunization. After the final blood sample collection, cats were challenged with FHV-1 SH-01c (10^6.5^ FFU/mL) and FCV GDJM-202201 (10^8^ FFU/mL). All cats were monitored for 14 consecutive days to record body temperature, body weight, survival status, and clinical manifestations; meanwhile, swabs were collected for viral load detection. (B-C) daily body weight and temperature measurements were recorded for each group on days post-immunization. (D-F) serum neutralizing antibody titers against FHV-1, FCV, and RABV were measured at pre-immunization and post-immunization time points. For each group, 5 serum samples were randomly selected for detection. Data are presented as mean ± SD (*n* = 5). Significant differences between groups were assessed using two-way ANOVA (*p < 0.05; **p < 0.01; ***p < 0.001; ****p < 0.0001; ns, not significant).

To evaluate the immunogenicity of the recombinant RABV vaccines in cats, the animals received three immunizations with rHEP-VP1-gB, rHEP-VP1-gD, or rHEP-Flury vaccines every two weeks. Blood samples were collected on days 7, 21, and 35. Subsequently, FCV and FHV-1 challenge assays were performed, followed by 14 days of monitoring changes in body weight and temperature, along with swab collection Figure 6(A). After the initial vaccination, daily monitoring of body weight and temperature changes was conducted. The results showed that post-immunization body temperatures remained within normal ranges, while body weights consistently increased Figure 6(B,C).

Neutralizing antibody assays revealed detectable but low levels of neutralizing antibodies against FCV in some cats of the rHEP-VP1-gB and rHEP-VP1-gD vaccine groups after the second immunization. However, a marked elevation in these titers was noted following the third immunization Figure 6(D). FHV-1 neutralizing antibodies were not detected in the rHEP-VP1-gB and rHEP-VP1-gD vaccine groups until after the third immunization Figure 6(E). Additionally, RABV neutralizing antibodies in some cats from rHEP-VP1-gB, rHEP-VP1-gD, and rHEP-Flury vaccine groups reached 0.5 IU/mL as early as 7 days after the first immunization. With subsequent booster immunizations, RABV neutralizing antibody titers in these groups increased significantly Figure 6(F).

## Challenge assay in cats

**Figure 7.**
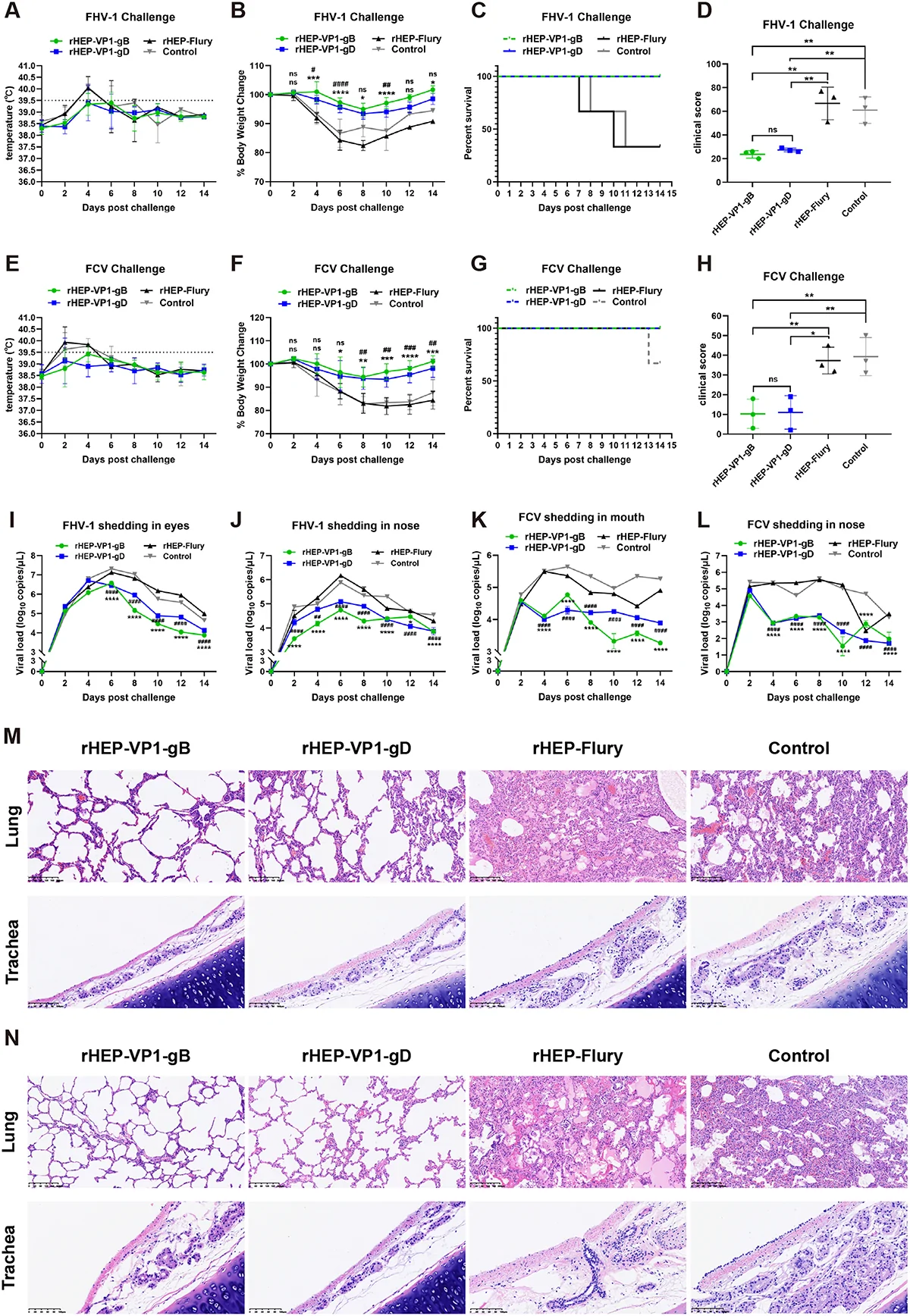
Challenge assay in cats. (A-D) following the FHV-1 inoculation, body temperature, body weight, survival status, and clinical manifestations of cats in each group were monitored and evaluated every 2 days for 14 consecutive days. (E-H) the aforementioned parameters were monitored and evaluated according to the same schedule post-inoculation with FCV. (I-J) ocular and nasal swabs were collected from cats in each group. Viral DNA was extracted, and FHV-1 load was quantified by qPCR. (K-L) oral and nasal swabs were collected from cats in each group. Viral RNA was extracted, reverse-transcribed into cDNA, and FCV load was detected via qPCR. (M-N) cats were necropsied after the 14-day challenge period. Lung and trachea tissues were collected for H&E staining, and representative images are shown. Scale bar: 200 μm. Data are presented as mean ± SD (*n* = 3). Significant differences between groups were assessed using *t*-test or two-way ANOVA. # statistical significance between the rHEP-VP1-gD and control groups; * statistical significance between the rHEP-VP1-gB and control groups (^#^or*p < 0.05; ^##^or**p < 0.01; ^###^or***p < 0.001; ^####^or****p < 0.0001; ns, not significant).

To further evaluate the protective efficacy of the recombinant RABV vaccines against FHV-1 and FCV, a challenge was performed on day 37 following the primary immunization. Cats in all groups developed fever by day 4 post FHV-1 challenge. In the rHEP-VP1-gB and rHEP-VP1-gD vaccine groups, some cats exhibited temperature exceeding 39.5°C, although none reached 40°C. In contrast, several cats in the rHEP-Flury vaccine and control groups recorded values above 40°C Figure 7(A). Cats in the rHEP-VP1-gB and rHEP-VP1-gD vaccine groups exhibited an initial body weight decline followed by a gradual increase after challenge. However, the magnitude of body weight loss in these groups was significantly less severe compared to that in the rHEP-Flury vaccine and control groups Figure 7(B). During the observation period, fatalities occurred in the rHEP-Flury vaccine and control groups, whereas no deaths were observed in the rHEP-VP1-gB and rHEP-VP1-gD vaccine groups Figure 7(C). The clinical manifestation scores were significantly higher in the rHEP-Flury vaccine and control groups compared to the rHEP-VP1-gB and rHEP-VP1-gD vaccine groups Figure 7(D).

After FCV challenge, cats in both the rHEP-Flury vaccine and control groups experienced fevers from day 2 to day 4, with some temperatures exceeding 40°C. In contrast, cats in the rHEP-VP1-gB and rHEP-VP1-gD vaccine groups exhibited milder fevers, with temperatures rising above 39.5°C but remaining below 40°C Figure 7(E). Body weight in all groups began to decline from day 4 post-challenge, then gradually recovered until day 10. Notably, cats in the rHEP-VP1-gB and rHEP-VP1-gD vaccine groups regained body weight more rapidly compared to those in the rHEP-Flury vaccine and control groups Figure 7(F). During the 14-day observation period, one fatality was recorded in the control group, while no deaths were observed in the rHEP-VP1-gB and rHEP-VP1-gD vaccine groups Figure 7(G). The clinical manifestation scores of the rHEP-VP1-gB and rHEP-VP1-gD vaccine groups were significantly lower than those of the rHEP-Flury vaccine and control groups Figure 7(H).

For FHV-1 shedding, viral loads in the ocular and nasal swabs of cats across all groups initially increased before declining, with viral nucleic acid remaining detectable in these swabs throughout the observation period. However, the rHEP-VP1-gB and rHEP-VP1-gD vaccine groups exhibited a marked reduction in viral load compared to the rHEP-Flury vaccine and control groups Figure 7(I,J). Similarly, the FCV load detected in oral and nasal swabs of cats in the rHEP-VP1-gB and rHEP-VP1-gD vaccine groups decreased significantly by day 4 post-challenge, followed by a gradual decline. The viral loads in the rHEP-VP1-gB and rHEP-VP1-gD vaccine groups were significantly lower than those in the rHEP-Flury vaccine and control groups Figure 7(K,L).

Histopathological analysis revealed that cats immunized with the rHEP-VP1-gB and rHEP-VP1-gD vaccines exhibited milder pathological damage in the lungs and trachea compared to those in the rHEP-Flury vaccine and control groups post FHV-1 Figure 7(M) and FCV Figure 7(N) challenge. Specifically, in the rHEP-Flury vaccine and control groups, the alveoli showed extensive inflammatory cell infiltration and epithelial cell hyperplasia, which collectively resulted in a marked thickening of the alveolar walls. Additionally, significant inflammatory cell infiltration was observed in both the mucosal and submucosal layers of the trachea. In contrast, the cats immunized with rHEP-VP1-gB and rHEP-VP1-gD vaccines showed only focal, mild inflammatory cell infiltration in alveoli and trachea submucosa.

## Discussion

According to the World Small Animal Veterinary Association feline vaccination guidelines, core vaccination against FCV, FPV, FHV-1, and RABV is strongly recommended for pet cats [29]. This recommendation underscores the significant clinical demand for multivalent feline vaccines, which directly aligns with the objective of the present research. Our previous studies have demonstrated that exogenous genes inserted between the *N* and *P* (or *G* and *L*) genes of the RABV genome can be efficiently expressed [26,30]. Furthermore, although rHEP-VP1-gB and rHEP-VP1-gD carrying two exogenous genes exhibited a delayed peak titer during *in vitro* culture, their maximum titer was comparable to that of the parental strain, which constitutes a favorable attribute for vaccine preparation. Given that RABV is highly pathogenic, strain safety is paramount for vaccine production; notably, the rHEP-VP1-gB and rHEP-VP1-gD exhibited pathogenicity comparable to that of the vaccine strain. Compared to traditional feline multivalent vaccines, the inactivated recombinant RABV vaccines developed herein not only maintain a favorable safety profile but also exhibit a simplified manufacturing process.

Although the recombinant RABV vaccines did not completely prevent clinical disease following FCV or FHV-1 challenge, vaccinated cats showed reduced clinical signs and lower viral shedding, indicating a certain degree of protection. It should be noted that currently available FCV and FHV-1 vaccines do not always provide sterilizing immunity [1,31]. The recombinant RABV-vector vaccine platform simultaneously provides antigens from RABV, FCV, and FHV-1 within a single vaccine construct. In addition to protection against FCV and FHV-1, these recombinant RABV vaccines also have the potential to induce protective immunity against rabies in cats. Given that rabies vaccination in cats is often less emphasized than in dogs in some regions [32], a multivalent vaccine strategy could improve rabies vaccine coverage.

To address the currently prevalent FCV strains in clinical settings, the FCV strain used in this study was isolated from a clinically infected cat in Jiangmen, China, ensuring relevance to local epidemiological conditions. It has been demonstrated that the VP1 protein can induce neutralizing antibodies and confer effective protection. For example, the CDE region of the VP1 protein, a major neutralizing epitope, can elicit neutralizing antibodies in cats and significantly alleviate the clinical symptoms of FCV infection [33]. Additionally, virus-like particles (VLPs) self-assembled from the VP1 protein can induce cross-neutralizing antibodies against diverse FCV strains, thereby conferring broad-spectrum protection across distinct genotypes [34,35]. A study has shown that a neutralizing antibody titer of at least 1:16 confers protection against heterologous FCV challenge, whereas a titer of ≤1:7 is insufficient to provide effective protection against heterologous variants [36]. However, a subsequent study has demonstrated that immunized cats with detectable antibodies, regardless of the magnitude of their antibody titers, exhibit a certain level of protection against FCV infection [37]. Furthermore, some studies have reported protection against virulent FCV challenge in cats with no detectable neutralizing antibodies, suggesting a role for cellular and innate immunity [38,39]. Therefore, it is essential to evaluate the immunization effects of the FCV vaccine from multiple perspectives. These findings highlight the complexity of protective immunity against FCV. FCV exhibits extensive genetic diversity, posing a major challenge for broadly protective vaccines [40,41]. Variations in the hypervariable region of VP1 reduce cross-protection among heterologous strains [42]. Therefore, broader protection likely depends on the selection of strains with broad immunogenicity and multivalent vaccine design [43,44]. In this context, recombinant RABV vaccine platforms expressing multiple antigens may help expand immune coverage.

Unlike the previous study [45], here we inserted two distinct exogenous genes at different loci within the recombinant RABV and compared the immunogenicity of the gB and gD antigens. However, the insertion sites were not further optimized, and this issue will be addressed in our subsequent research. Glycoproteins gD, gB, and gC play essential roles in mediating viral attachment to host cellular receptors and facilitating entry into susceptible cells [46–49]. Previous studies have demonstrated that these glycoproteins can serve as vaccine antigens, as they can elicit the production of neutralizing antibodies against FHV-1 [50–53]. The neutralizing antibody titer induced by gB protein expressed on bacterial particles was similar to that induced by gD protein, while the titer elicited by gC protein was the lowest among the three [15]. Therefore, gB and gD were selected as the research targets in the present study. Regarding FHV-1 vaccine efficacy, previous studies have reported that an inactivated FHV-1 vaccine induced a mean neutralizing antibody titer of 1:45.5 in immunized cats [54]. Furthermore, although FHV-1 neutralizing antibody titer induced by the recombinant live virus vaccine was less than 1:4, the immunization conferred protective efficacy against FHV-1 challenge [55]. However, neutralizing antibody levels alone cannot fully reflect a vaccine’s protective efficacy. For instance, an FHV-1 modified live virus vaccine failed to induce detectable neutralizing antibodies in SPF kittens, yet subsequent challenge assays demonstrated partial protective effects despite the absence of measurable antibodies [56]. Consistent with these observations, although relatively low neutralizing antibody titers against FCV and FHV-1 were detected in the present study, vaccinated cats exhibited reduced clinical signs and lower viral shedding following challenge. These findings suggest that protective immunity may not be fully reflected by pre-challenge neutralizing antibody titers alone and that vaccine-induced immune memory and other immune mechanisms may contribute to protection.

A limitation of this study is that it did not investigate the specific molecular conformations of the gB, gD, and VP1 proteins as vaccine antigens. Drawing on previous research [16,57], we propose that the gB and gD proteins may be incorporated into the viral envelope during viral budding, while the VP1 protein may self-assemble into VLPs. These hypotheses require further experimental validation. Another limitation of the present study is the relatively small number of cats included in each experimental group, which may limit the statistical power of the findings. Statistical analyses were intended as exploratory assessments and should therefore be interpreted with caution. In addition, although all cats were screened by PCR and selected based on FCV- and FHV-1-neutralizing antibody titers below 1:2 prior to enrollment, low levels of maternally derived antibodies and undetected prior FHV-1 exposure cannot be completely excluded [13]. These factors could influence vaccine-induced immune responses in kittens [58]. Further studies with larger animal cohorts and more comprehensive virological and immunological characterization are warranted to confirm the reproducibility and protective efficacy of the recombinant RABV vaccines.

In addition to the above limitations related to immunological evaluation and sample size, another limitation of this study lies in the histopathological analysis. Although FCV and FHV-1 primarily replicate in upper respiratory tract tissues, including the nasal mucosa and conjunctival epithelium [59,60], necropsy-based studies have reported that, in severe cases, infection can extend to the lower respiratory tract, where lesions such as pneumonia can occur in the trachea and lungs [61]. Accordingly, lung and tracheal tissues were selected for histopathological analysis. However, the absence of upper respiratory tract tissues, such as nasal turbinate or conjunctiva, represents a limitation of the present study. Future studies should investigate pathological changes and viral distribution in these tissues to provide a more comprehensive evaluation of vaccine protective efficacy.

In summary, two inactivated recombinant RABV vaccines were constructed in this study. These vaccines exhibited favorable safety profiles, elicited robust neutralizing antibody responses against FHV-1, FCV, and RABV, and conferred effective protective immunity to animals challenged with these pathogens. Based on these findings, the RABV vector platform demonstrates substantial potential for the development of a quadrivalent vaccine targeting FCV, FPV, FHV-1, and RABV.

## Supplementary Material

Supplementary_Tables_S1_to_S6 (1).docx

Supplementary Figure S1.docx

## Data Availability

The raw data supporting this study’s findings are available in Science Data Bank at https://doi.org/10.57760/sciencedb.33741 [62].
