# Supplementary material for: Immunogenicity evaluation of recombinant rabies virus-vectored multivalent vaccines expressing feline calicivirus VP1 and feline herpesvirus 1 gB/gD genes
Source: Virulence. 2026 Sep 7;17(1):2728426. doi: 10.1080/21505594.2026.2728426 (PMC13556961; doi:10.1080/21505594.2026.2728426)
Supplement: Supplementary file 1 [file KVIR_A_2728426_SM2255.docx]

**Figure S1**

**Figure S1**. Western blot validation of the specificity of polyclonal antibodies against FCV VP1 and FHV-1 gB and gD proteins. (A) Crandell-Rees feline kidney (CRFK) cells were infected with FCV at an MOI of 0.1, and cell lysates collected at 24 h post-infection were probed with a polyclonal antibody against VP1. (B, C) CRFK cells were infected with FHV-1 at an MOI of 0.1, and cell lysates collected at 24 h post-infection were probed with polyclonal antibodies against gB and gD, respectively. Lysates from mock-infected CRFK cells were included as negative controls.
