# Supplementary material for: Immunogenicity evaluation of recombinant rabies virus-vectored multivalent vaccines expressing feline calicivirus VP1 and feline herpesvirus 1 gB/gD genes
Source: Virulence. 2026 Sep 7;17(1):2728426. doi: 10.1080/21505594.2026.2728426 (PMC13556961; doi:10.1080/21505594.2026.2728426)
Supplement: Supplementary file 2 [file KVIR_A_2728426_SM2256.docx]

**Supplementary Table S1. Primers used for amplification of the *VP1*, *gB*, and *gD* genes for construction of recombinant RABVs.**

| Primer | Sequence (5'-3') | Purpose |
| --- | --- | --- |
| F-VP1 | *CGTACAATTCTGCAGTTTGC*ATGGCTGATGATGGCTCAATTACC | Amplification of FCV *VP1* gene inserted between the *N* and *P* genes of the RABV genome |
| R-VP1 | *CAAGATGCATCATACAAGAA*TCATAATTTGGTCATAGAGCT |  |
| F-gB | *AAAAACTAACACCTCTCGTACG*ATGTCCACTCGTGGCGATCTTG | Amplification of FHV-1 *gB* gene inserted between the *G* and *L* genes of the RABV genome |
| R-gB | *AGTTATGTACCGGTCCTG*TTAGACAAGATTTGTTTCAGTATCATCACCGC |  |
| F-gD | *AAAAACTAACACCTCTCGTACG*ATGATGACACGTCTACATTTTTGGTGG | Amplification of FHV-1 *gD* gene inserted between the *G* and *L* genes of the RABV genome |
| R-gD | *AGTTATGTACCGGTCCTG*TTAAGGATGGTGAGTTGTATGTATTATAGG |  |

Note: Italicized sequences indicate homologous arms.

**Supplementary Table S2. Primers used for amplification of the pHEP3.0 vector.**

| Primer | Sequence (5'-3') | Purpose |
| --- | --- | --- |
| F-pHEP3.0 | TTCTTGTATGATGCATCTTGAAAAAAACTAACACTCCTCCTTTCGAACCATCCCAAGTATG | Amplification of pHEP3.0 plasmid backbone for linearization and introduction of TIS and TTS sequences |
| R-pHEP3.0 | GCAAACTGCAGAATTGTACGAGAGGTGTTAGTTTTTTTCATGATGGATATACACAATCTATGGATTTCTGGC |  |

**Supplementary Table S3. Primers used for amplification of the *VP1*, *gB*, and *gD* genes for construction of prokaryotic expression plasmids.**

| Primer | Sequence (5'-3') | Purpose |
| --- | --- | --- |
| F-VP1-28a | *GTGGACAGCAAATGGGTCGC*GATGATGGCTCAATTACCACACCAG | Amplification of FCV *VP1* gene for cloning into the pET-28a vector |
| R-VP1-28a | *CAGTGGTGGTGGTGGTGGTG*TAATTTGGTCATAGAGCTCCTAATG |  |
| F-gB-32a | *ACAAGGCCATGGCTGATATC*ATCTGGATCGTACTATTCTTAGTCGG | Amplification of the FHV-1 *gB* gene for cloning into the pET-32a vector |
| R-gB-32a | *GTGCTCGAGTGCGGCCGC*TAAGGCGTGGAGTTGGTTGC |  |
| F-gD-32a | *ACAAGGCCATGGCTGATATC*ATGATGACACGTCTACATTTTTGG | Amplification of the FHV-1 *gD* gene for cloning into the pET-32a vector |
| R-gD-32a | *GTGCTCGAGTGCGGCCGC*TACTGACTGATTATTCATGCCC |  |

Note: Italicized sequences indicate homologous arms.

**Supplementary Table S4. Primers used for PCR detection of FCV and FHV-1.**

| Primer | Sequence (5'-3') | Purpose |
| --- | --- | --- |
| F-FHV | ATGACACGTCTACATTTTTGG | Detection of FHV-1 *gD* gene |
| R-FHV | TACTGACTGATTATTCATGCCC |  |
| F-FCV | ATGAGCTCAACTTGCGCTAACG | Detection of FCV *VP1* gene |
| R-FCV | GGCGGTTTCAAAAGGTGGAAC |  |

**Supplementary Table S5. Primers used for qPCR quantification of FCV and FHV-1 viral loads.**

| Primer | Sequence (5'-3') | Purpose |
| --- | --- | --- |
| F-qP-FCV | ACACATTGCTGCTCACATCC | Detection of FCV ORF1 region for viral load quantification |
| R-qP-FCV | CTCTCCATTCAATACGAGCAGC |  |
| F-qP-FHV | ATGGAGCTCGTGATTACCAC | Detection of FHV-1 *gD* gene for viral load quantification |
| R-qP-FHV | TTGGACCGTTTCGAATCCTC |  |

**Supplementary Table S6. Primers used for junction PCR detection of foreign gene insertions at the N–P and G–L junctions of recombinant RABVs.**

| Primer | Sequence (5'-3') | Purpose |
| --- | --- | --- |
| F-NP | CAGATGACGGAACTGTCAAC | Detection of foreign gene insertion at the N–P junction region of recombinant RABV (junction PCR) |
| R-NP | AAGTCCTCTCGATACTTGCC |  |
| F-GL | AAAGCAATCTTGGAGGGAC | Detection of foreign gene insertion at the G–L junction region of recombinant RABV (junction PCR) |
| R-GL | GTCATCATAAACCTCTCCC |  |
